# Monitoring the Evaporation of Fluids from Fiber-Optic Micro-Cell Cavities

**DOI:** 10.3390/s131115261

**Published:** 2013-11-07

**Authors:** Eyal Preter, Borut Preloznik, Vlada Artel, Chaim N. Sukenik, Denis Donlagic, Avi Zadok

**Affiliations:** 1 Faculty of Engineering, Bar-Ilan University, Ramat-Gan 52900, Israel; E-Mail: pre_eyal@yahoo.com; 2 Faculty of Electrical Engineering and Computer Science, University of Maribor, 2000 Maribor, Slovenia; E-Mails: borut.preloznik@uni-mb.si (B.P.); ddonlagic@uni-mb.si (D.D.); 3 Department of Chemistry, Bar-Ilan University, Ramat-Gan 52900, Israel; E-Mails: vlada2009@gmail.com (V.A.); Chaim.Sukenik@biu.ac.il (C.N.S.)

**Keywords:** fiber-optic sensors, opto-fluidics, evaporation monitoring, optical micro-cells, fiber cavities, droplet analysis

## Abstract

Fiber-optic sensors provide remote access, are readily embedded within structures, and can operate in harsh environments. Nevertheless, fiber-optic sensing of liquids has been largely restricted to measurements of refractive index and absorption spectroscopy. The temporal dynamics of fluid evaporation have potential applications in monitoring the quality of water, identification of fuel dilutions, mobile point-of-care diagnostics, climatography and more. In this work, the fiber-optic monitoring of fluids evaporation is proposed and demonstrated. Sub-nano-liter volumes of a liquid are applied to inline fiber-optic micro-cavities. As the liquid evaporates, light is refracted out of the cavity at the receding index boundary between the fluid and the ambient surroundings. A sharp transient attenuation in the transmission of light through the cavity, by as much as 50 dB and on a sub-second time scale, is observed. Numerical models for the transmission dynamics in terms of ray-tracing and wavefront propagation are provided. Experiments show that the temporal transmission profile can distinguish between different liquids.

## 1. Introduction

Evaporation is the transition from the liquid phase to the gas phase that occurs at temperatures below the boiling point, typically at ambient pressure [1]. Evaporation is related to properties of the fluid itself, the ambient environment and the surface on which the fluid is applied, and has diverse potential applications such as in climatography [2], cooling of electronic circuits [3] and even medical analysis of tears in the eyes [4]. Analysis of the formation, geometry and evaporation of fluid droplets can provide information on numerous properties of liquids including surface tension, viscosity, refractive index and chemical composition of solutions. In most cases, droplets are monitored through direct observation in contact angle goniometers, by micro-gravimeters, by atomic force microscopy, or using measurements of electrical capacitance [5–8]. These methods provide high accuracy, however they often require sophisticated auxiliary equipment in the immediate vicinity of the fluid droplet.

Optical fibers constitute an exceptional sensing platform [9]. They provide remote access to harsh environments, can be readily embedded within structures, and may serve as minimally-intrusive, bio-compatible probes. Fiber optic sensors could offer simple, low-cost solutions for evaporation monitoring. The commonly observed quantities in fiber-optic fluid sensors are either refractive index or absorption spectrum. These observables provide rather limited information on evaporation dynamics and droplet behavior. Salazar-Haro and coauthors measured the reflection spectrum of a static droplet on the tip of an optical fiber to analyze the droplet geometry [10]. McMillan *et al.* used optical fibers in a capillary configuration in the monitoring of droplet formation [11].

In this work, we demonstrate the monitoring of the evaporation dynamics of sub-nano-liter fluid volumes, from within in-line micro-cavities that are etched through optical fibers. These all-fiber-based silica cavities, or micro-cells (MCs), offer unique advantages such as potential for remote access and for spatial multiplexing, use over a broad temperature range, chemical inertness and small dimensions [12]. Accordingly, fiber MCs have been utilized as miniature sensors of a fluid's refractive index [12–14], mechanical strain [15–17] and temperature [18,19]. However, we find no report of the monitoring of fluid evaporation using MCs.

The sensing principle relies on the temporally-varying refraction of light out of the MC. The propagation loss through an MC that is uniformly filled, with either fluid or air, is comparatively low, on the order of a few dB. As the fluid evaporates from within the MC, however, a refractive index boundary is formed between the receding fluid and the ambient air. As the index discontinuity crosses the path of light through the MC, a substantial fraction of the incident optical power is refracted out of the MC. The refraction occurs on a sub-second time scale, and it is associated with temporary propagation losses of as much as 50 dB. The duration and details of the loss profile, in turn, depend on the rate of evaporation of the fluid, and on the specific geometry it assumes within the MC during the process. Simple measurements of propagation loss could provide, therefore, a signature of a fluid under test and/or the environmental conditions. The signature is governed by physical properties such as boiling point, vapor pressure, surface tension, strength of adsorption onto the silica walls *etc.*, and is not directly related to the refractive index or the absorption spectrum. Hence the sensing principle potentially provides a new approach to the fiber-optic recognition and analysis of fluids.

We have modeled the propagation of light through the MC during fluid evaporation using both ray-tracing and wavefront propagation analysis. The results of the simulations correlate well with the experimentally observed temporal losses. Based on measurements of the transient propagation losses, we were able to distinguish between ethanol, acetone and hexane as sample test liquids, and to recognize a mixture of ethanol and hexane. Preliminary results of our work have been reported [20,21]. Section 2 below provides a detailed description of the principle of operation of the sensors and describes the modeling of propagation loss dynamics. Experimental results are provided in Section 3, and a concluding discussion is given in Section 4.

## 2. Principle of Operation and Modeling

**Figure 1.**
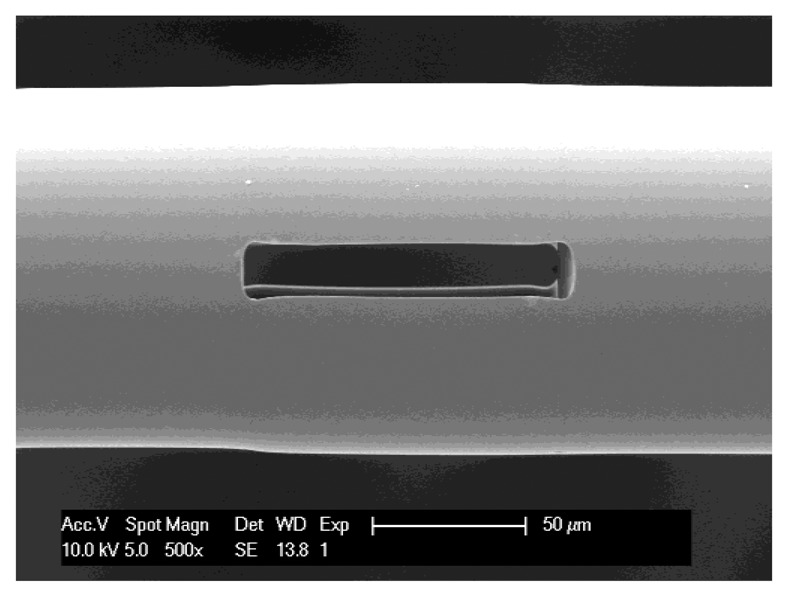
Scanning electron microscope image of a 100 μm-long, all-fiber MC used in the monitoring of fluid evaporation.

Figure 1 shows a scanning electron microscope image of the 100 μm-long MC used in the monitoring of evaporation. The MCs are fabricated by selectively etching a segment of specialty fiber, which is spliced in between two sections of standard single-mode fiber [12]. A highly elliptic region at the center of the specialty fiber is doped with ∼8.7% phosphorus pentoxide (P_2_O_5_) [12]. The doped region etches in hydrofluoric acid (HF) at a rate that is approximately 35 times higher than that of pure silica [22]. Hence, when the HF reaches the doped region it removes this region at a high rate, resulting in the narrow, elongated, cavity seen in Figure 1.

The modeling of light propagation through the MC during the evaporation of a fluid droplet is based on several assumptions. First, the geometry of the problem is reduced to two dimensions. This reduction is in agreement with the geometry of the MC, which is highly non-symmetric in the azimuthal direction. Light propagates along the fiber axis *z*, and the analysis is restricted to the *xz* plane, where *x* is the transverse direction along which the fiber is etched.

Next, the instantaneous spatial profile of the droplet during the process of evaporation has to be specified. Subject to ideal conditions, there is no physical preference to evaporation through either side of the MC. In particular, gravity is negligible in comparison to the inter-molecular forces for small liquid volumes. Nevertheless, direct observation of the evaporation process suggests that symmetry is not maintained. Instead, evaporation is initiated from one side of the MC, due to small-scale disturbances and instabilities such as air flow, geometry variation and local surface roughness. Asymmetric droplet profiles are characterized by a single refractive index boundary between fluid and air. Two specific asymmetric droplet geometries were used in simulations. Table 1 describes the two profiles, in terms of the x-axis position *X* of the index boundary for given position *z* and time *t*.

**Figure 2.**
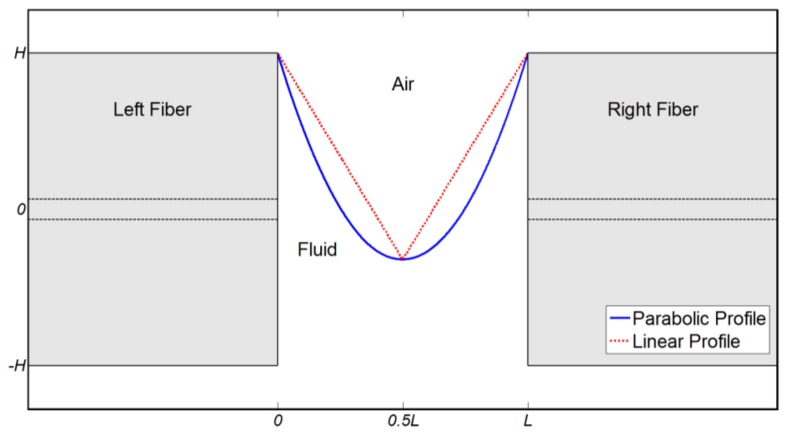
Examples of the boundaries between fluid and air in the *xz* plane within a MC, during the evaporation of the fluid, as used in simulations. Blue, solid curve: parabolic droplet profile, *O*(*t*) = −20 μm (see Table 1). Red, dashed line: linear profile, *O*(*t*) = −20 μm, (see Table 1). Black, dashed lines: cores of the fibers at the input and output of the MC.

In the equations in Table 1, *L* = 100 μm is the MC length, *H* = 62.5 μm denotes the fiber radius, and *O*(*t*) is the height of the index boundary at *z* = *L*/2 (or the minimum height of the fluid within the MC), at instance *t*. The temporal evolution of *O*(*t*) defines the progress of evaporation. Note that both profiles are symmetric along the fiber axis, with respect to the center *z* = *L*/2. Figure 2 shows examples of the boundaries between fluid and air for the two profiles.

### 2.1. Ray-Tracing Analysis

**Figure 3.**
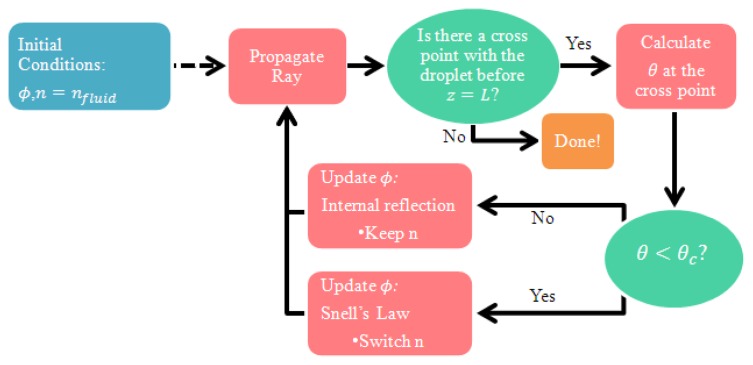
Flow chart of ray tracing simulations. *n*: Refractive index. *n_fluid_*: Refractive index of the fluid within the MC (taken as 1.358, the refractive index of ethanol at 1,550 nm wavelength and in room temperature). *ϕ*: Angle between the direction of propagation of a ray and the fiber axis. *θ*: Angle of incidence between the direction of propagation of a ray, and the normal to the boundary between fluid and air. *θ*_c_: Minimum angle of incidence for which total internal reflection takes place at the boundary between fluid and air.

The geometries of the fluid droplets within the MC were used first in simulations of rays tracing. Rays are emitted from the core region of the single mode fiber leading into the MC from the left-hand side (*z* = 0), at a range of possible angles within the numerical aperture (*NA*) of the fiber in fluid. The *NA* of a single mode fiber in ethanol (refractive index of 1.358, at 1,550 nm wavelength and in room temperature), is 0.12. Following multiple reflections and refractions at the interfaces between fluid and air, each ray reaches the opposite end of the MC (*z* = *L*), where it may or may not couple into the core of the output single mode fiber. Figure 3 shows the ray tracing simulation flow chart. A similar analysis has been reported by McMillan *et al.* [11].

**Figure 4.**
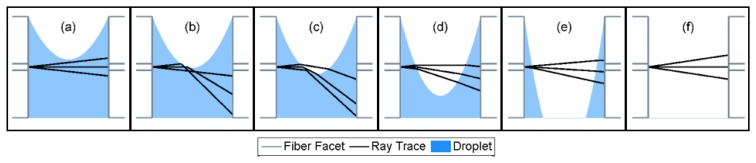
Calculated ray-tracing during the evaporation of fluid from within a MC. An initial phase of low propagation losses (**a**) is followed by an intermediate phase of large loss (**b**–**d**), as reflected rays do not reach the core of the output fiber. Transmitted power recovers gradually towards the end of the evaporation process (**e**–**f**). The values of *O*(*t*) for panels (a–f) were 9.5 μm, −1 μm, −8.5 μm, −35 μm, −110 μm, and O(t) →∞, respectively.

Supplementary Materials Item 1 shows a video of the simulated ray tracing dynamics during the evaporation process. Figure 4 shows snapshots of the simulation video, taken at several time points. Three rays are included in simulations, corresponding to initial values of *ϕ* = 0, and *ϕ* = ±*NA* = ±0.12. The movie shows that in the beginning of the evaporation process (Figure 4a), the rays are not affected by the receding fluid boundary. The simulations therefore suggest a relatively low propagation loss in the initial phase of evaporation. As the evaporation proceeds, however, the rays intersect the fluid front, undergo a total internal reflection, and do not reach the right hand fiber core area (Figure 4b,c). High propagation losses are anticipated during these intermediate stages. As the fluid recedes further, total internal reflection gradually gives way to refraction according to Snell's law (Figure 4c,d). The collected power at the output end of the MC is expected to gradually recover at this stage, reaching that of an air-filled cavity when evaporation is completed (Figure 4e,f).

### 2.2. Wavefront Propagation Analysis

Ray tracing analysis is comparatively simple, and provides useful intuition for the dynamics of propagation loss through the MC during fluid evaporation. However, it is difficult to provide a quantitative prediction for the expected losses based on ray tracing. Therefore a second simulation was carried out, in terms of wavefront propagation through the MC. The wavefront at the input end of the MC at *z* = 0 is that of the fiber mode, and it is well approximated by a Gaussian transverse profile *E_G_*(*x*) with a spot size radius of 4.2 μm. The wavefront is propagated to the center of the non-uniform MC medium *z* = *L*/2, based on the Huygens–Fresnel principle [23]: each point *A_i_* of the input Gaussian field profile is regarded as a point source of a spherical wave with a wavelength *λ*, so that:

(1) $$E_{L/2}^{\text{left}}\left(u_{j}\right)=C_{0}\cdot \sum_{i=-\infty }^{\infty }\frac{1}{r_{ij}}E_{G}\left(U_{i}\right)e^{-ik_{0}r_{ij}}$$

**Figure 5.**
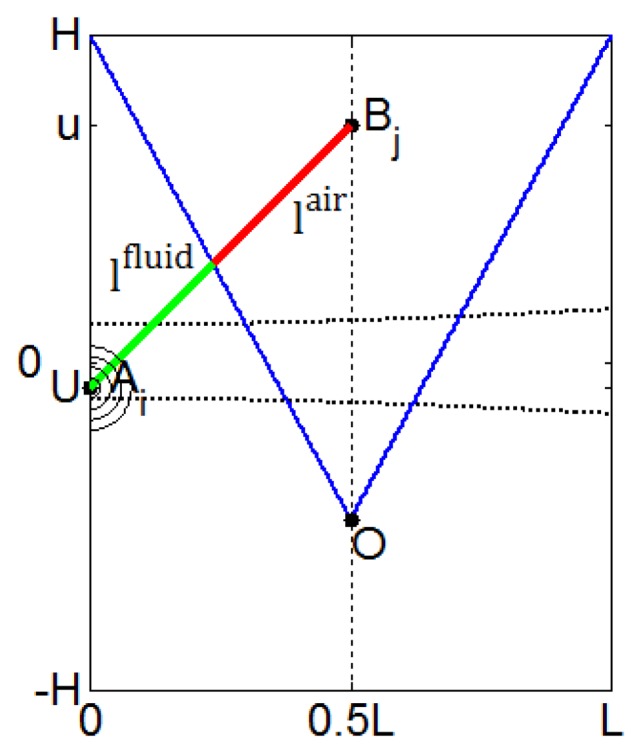
Huygens–Fresnel analysis of the propagation of light in a MC that is partially filled with fluid. A linear droplet profile is assumed (blue solid line, see Table 1). *A_i_* is a point source within the wavefront that is emitted from the left-hand fiber. The wavefront is well approximated by a Gaussian beam. The width of that beam in a uniform fluid medium, as a function of *z*, is denoted by the black dashed lines. *B_j_* is an observation point in the plane *z* = *L*/2, where the field is calculated. The distance from *A_i_* to *B_j_* is comprised of a segment of propagation in fluid (green), and a segment of propagation in air (red).

In Equation (1), the field distribution at a point *B_j_* in the observation plane is denoted by $E_{L/2}^{\text{left}}\left(u_{j}\right)$, *u_j_* and *U_i_* are the *x* -direction coordinates of *A_i_* and *B_j_* respectively, *k_0_*= 2*π*/*λ*, and *C*_0_ is a normalization constant. The distance between *A_i_* and *B_j_* is given by $R_{ij}\equiv \sqrt{{\left(0.5L\right)}^{2}+{\left(U_{i}-u_{j}\right)}^{2}}$. This distance is the sum of a length of propagation in fluid $l_{ij}^{\text{fluid}}$, and a length of propagation in air $l_{ij}^{\text{air}}$, which can both be found with knowledge of the droplet profile at a given instance *t* of interest (see Figure 5 and Table 1). Lastly, the optical path length *r_ij_* is given by $r_{ij}=n_{\text{fluid}}\cdot l_{ij}^{\text{fluid}}+l_{ij}^{\text{air}}$. The analysis does not account for the change in direction of propagation at the refractive index boundary between fluid and air.

**Figure 6.**
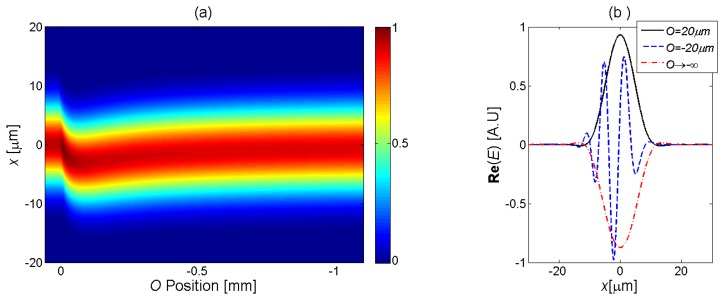
(**a**) Calculated normalized transverse profile ${\left|E_{L/2}^{\text{left}}\left(u_{j}\right)\right|}^{2}$ of the propagating wavefront at the center of the MC *z* = *L*/2, as a function of the height *O* (*t*) of the fluid droplet at the center of the MC. A linear droplet profile was used in the simulation (see Table 1). (**b**) Normalized real part of $E_{L/2}^{\text{left}}\left(u_{j}\right)$, calculated for three values of *O* (*t*): 20 μm (black solid curve), −20 μm (blue dashed curve), and *O* (*t*) → −∞ (red dash-dotted curve).

Figure 6a presents the calculated transverse distribution of the field at the center of the MC $E_{L/2}^{\text{left}}\left(u_{j}\right)$ during evaporation, as a function of the droplet geometry parameter *O* (*t*). A linear droplet shape was used in the simulation. The field cross-section initially follows the diffraction pattern of a Gaussian beam. As the evaporation progress continues, and *O* (*t*) approaches the transverse extent of the beam, the wavefront gradually becomes tilted and offset downwards. In addition, the phase of the waveform becomes distorted, as seen by the oscillations in the real part of $E_{L/2}^{\text{left}}\left(u_{j}\right)$ (Figure 6b). The tilt and distortion of the wavefront would manifest in high transmission losses. The wavefront at the center of the MC is gradually restored to a centered Gaussian profile with a uniform transverse phase, as the fluid disappears completely (large negative values of *O* (*t*)).

The transmission loss through the MC can be obtained, in principle, through the further propagation of $E_{L/2}^{\text{left}}\left(u_{j}\right)$ to the output facet of the MC at *z* = *L*, and calculation of the overlap integral between the resulting field profile and that of the fiber mode. Instead, we found it more convenient to back-propagate the transverse profile of the fiber mode from *z* = *L* to z = *L*/2, in a similar manner to that of Equation (1), resulting in a transverse field profile denoted as $E_{L/2}^{\text{right}}\left(u_{j}\right)$. The relative transmission of power through to the fiber mode at the MC output is calculated through the overlap integral (assuming normalized field profiles):

(2) $$\mathrm{Loss}\left[O\left(t\right)\right]={\left|\int \;E_{L/2}^{\text{left}}\left(u_{j}\right)\;\cdot \;{\left[E_{L/2}^{\text{right}}\left(u_{j}\right)\right]}^{*}du_{j}\right|}^{2}.$$

**Figure 7.**
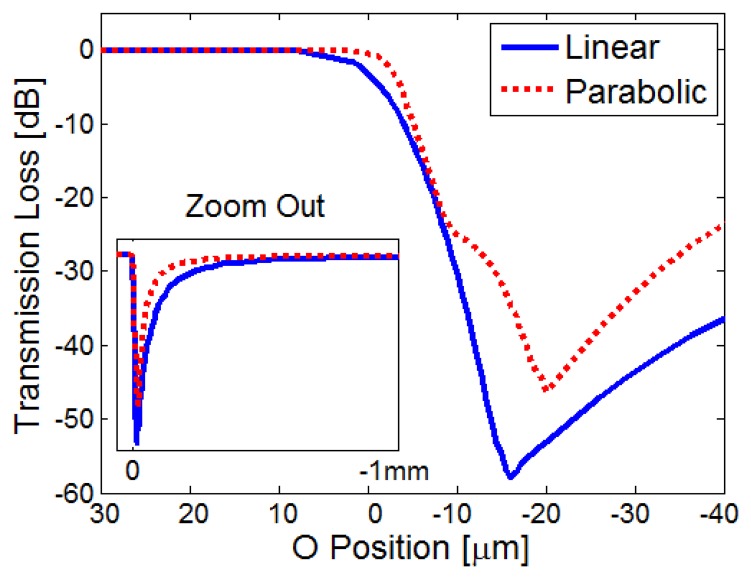
Calculated transmission losses through a MC during the evaporation of a fluid, as a function of the height *O* (*t*) of the fluid droplet at the center of the MC (*z* = *L*/2). Blue, solid curve: linear droplet profile (see Table 1). Red, dashed curve: parabolic droplet profile (see Table 1). The inset shows the transmission loss curve over a larger range of *O* (*t*) values.

Figure 7 shows the calculated transmission losses through the MC as a function of *O* (*t*). Both linear and parabolic droplet profiles were simulated. For both profiles, the transmitted power begins to decrease when the droplet minimum point *O* (*t*) reaches the transverse extent of the beam. In the linear (parabolic) profile, the maximal loss is −58 dB (−46 dB), obtained when *O* (*t*) equals −16 μm (−20 μm). From that instance of maximum loss, the transmitted power recovers monotonously until it reaches its steady-state value, corresponding to an air-filled MC (*O* (*t*) → −∞).

The simulation results in Figure 7 suggest that very large temporary losses, on the order of 50 dB, can be expected during the evaporation of fluids from within MCs. This agrees with the qualitative trend that was obtained using ray tracing. Simulations also suggest that the details of the loss transients may vary with the specific geometric profile assumed by the fluid droplet inside the MC, and with the rate in which the refractive index boundary is receding. Since different fluids are expected to evaporate at different rates, and display a different balance between their own surface tension and their attachment to the silica walls, we may expect that the monitoring of loss transients could distinguish between them.

## 3. Experimental Setup and Results

Light from a 1 mW laser diode at 1,548 nm wavelength was used in transmission loss measurements. The optical power at the output of the MC was monitored by a low-bandwidth power meter and an oscilloscope. The state of polarization of the incoming light was set for maximum power transmission. A few pico-liters droplets of different volatile organic liquids were applied to the MC in a repeatable manner. All tested liquids caused complete wetting of the MC, so that the liquids were initially in contact with the inner cavity walls. Experiments were carried out successively under ambient laboratory conditions.

**Figure 8.**
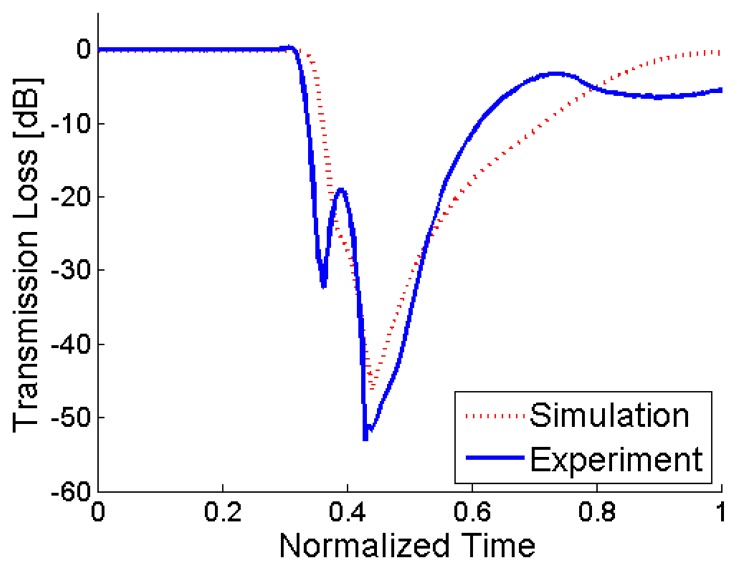
Transmission loss during ethanol evaporation from within a MC: experiment (blue, solid) and simulation (red, dashed). A parabolic droplet profile was used in the simulations. The times scales of the two curves were normalized and aligned so that the transmission minima coincide, and the durations of the loss transients match.

Figure 8 shows the transmitted optical power as a function of time during the evaporation of ethanol from within a 100 μm-long MC [20]. The collected power falls initially by some 30 dB, temporary increases by about 10 dB, and decreases again to approximately 50 dB below initial values, before returning to the transmission loss values of an air-filled MC. The simulated loss transient, as calculated using wavefront propagation and a parabolic droplet profile, is shown in the figure as well. Our previous work had shown that evaporation takes place at a constant rate [21], hence a linear time dependence of *O* (*t*) was assumed in the model. The time scales of both curves were normalized and aligned so that the transmission minima coincide and the durations of the loss transients match. The general trends in the curves are in good agreement: an initial low loss, followed by pronounced temporal attenuation and a gradual recovery of the transmitted power. However, in the experiment a secondary local transmission maximum is observed, and the steady state loss values towards the end of the evaporation process differ. Those differences are attributed to several factors, mainly to the dimensional reduction in the modeling, and to the arbitrary selection of the fluid droplet profile in the simulations. Other potential causes for differences are mechanical defects such as local roughness variations of the MC, and impurities of the liquid.

**Figure 9.**
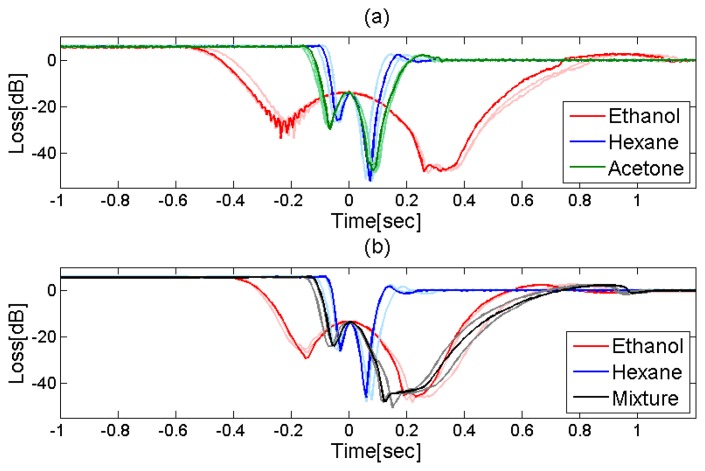
Experimentally measured, relative transmission power losses as a function of time, recorded during the evaporation of acetone (green), ethanol (red), hexane (blue), and a 1:1 mixture of ethanol and hexane (black), from within a fiber MC. Colors relate to both panels. In order to facilitate a comparison between the loss transients of different solvents, the origin *t* = 0 for each curve was arbitrarily chosen as the instance of the local maximum transmission, which occurs during each loss event.

Figure 9a shows the transmitted optical power as a function of time, during the evaporation of ethanol, acetone and hexane from within the same MC used in Figure 8 [20]. All transmission curves are characterized by sharp attenuation transients. The duration of the loss transient is fluid-specific: approximately 0.2 s for hexane, 0.3 s for acetone, and 1 s for ethanol. The temporal loss profiles are highly repeatable, as can be seen in both panels of Figure 9. Three different curves are presented for each solvent, all showing similar transients. The small-scale variations stem from impurities of the fluid and small fluctuations in the environmental conditions. The differences among the transmission profiles of the three solvents are distinct and reproducible.

In a similar experiment, a 1:1 mixture of ethanol and hexane was introduced into the MC. Figure 9b compares the transmission transients that accompany the evaporation of the two pure liquids and their mixture. The attenuation event recorded for the mixture is of an intermediate duration, in between that of hexane and that of ethanol. Furthermore, the leading edge in the mixture attenuation profile follows that of hexane, whereas the trailing edge is reminiscent of that of ethanol, which is slower to evaporate [20].

**Figure 10.**
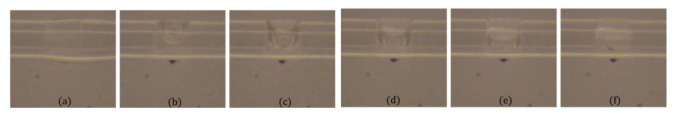
Microscope images of the MC: (**a**): filled with ethanol; (**b**–**e**): during the evaporation of ethanol; (**f**): upon the completion of evaporation. The transmission loss of 1,548 nm wavelength light was monitored in synchronization with the microscope images. Large losses were observed when the MC was partially filled.

**Figure 11.**
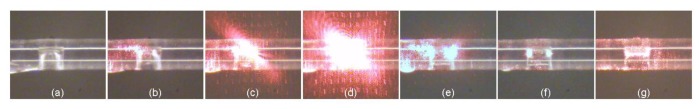
Microscope images of the MC: (**a**): filled with ethanol; (**b**–**f**): during the evaporation of ethanol; (**g**): upon the completion of evaporation. Red light was propagated through the MC during the evaporation of the ethanol droplet. Large refraction of light out of the MC is observed when it is partially filled.

The association of pronounced transient transmission losses with refraction at the receding boundary between refractive indices was further verified in several control experiments, which included direct observation of the MC through an optical microscope, synchronized with the output power measurements. Figure 10 shows snapshots from a video file, taken by the camera of the optical microscope during the evaporation of an ethanol droplet. Panel (a) shows a fluid-filled MC, panels (b–e) represent images captured during evaporation, whereas panel (f) shows an air-filled MC. High transmission loss coincided with the evaporation transition, in which the micro-cell is only partially filled with fluid. Supplementary Materials Item 2 shows a microscope video file, in which red light was used at the MC input instead of the telecommunication wavelength source. Figure 11 shows snapshots images from the video file, in which strong refraction of light out of the MC is evident. As before, panel (a) represents a fluid-filled MC, panels (b–f) show successive frames taken during the evaporation, and in the last panel (g) the MC is air-filled.

## 4. Summary

A new concept for the fiber-optic recognition and analysis of fluids is proposed, analyzed and experimentally demonstrated. A sub-nano-liter droplet of a volatile fluid under test is applied to an inline, all-fiber MC. The evaporation of the droplet is accompanied by large, transient losses in the transmission of light through the fiber MC. Losses occur on sub-second scale durations and may reach 50 dB, and are therefore simple to observe and record. The losses stem from pronounced refraction of light out of the MC, which takes place as the receding index boundary between the evaporating fluid and the ambient air crosses the light path. The loss mechanism is identified in direct observation control experiments, and is supported by ray-tracing and wavefront propagation simulations.

The detailed dynamics of the transmission loss transients vary with the rate of evaporation, and with the specific geometry of the droplet inside the MC. These, in turn, depend on physical attributes of the fluid, such as its boiling point, vapor pressure, surface tension, and the strength of the bonds formed between its molecules and the MC silica walls. Transmission loss measurements may therefore distinguish between different fluids, and/or recognize changes in environmental conditions. In the experiments, fiber-optic evaporation monitoring was shown to distinguish between samples of ethanol, acetone, hexane, and 1:1 mixtures of ethanol and hexane. The analysis can be extended to a quantitative measurement of relative concentrations in binary mixtures. The evaporation of mixtures is known to consist of several phases, and the evaporation dynamics in each varies with the relative concentrations [24,25]. We can therefore calibrate our sensor and observe distinct temporal loss profiles for different concentrations.

All tested liquids, as well as the vast majority of organic solvents in general, caused complete wetting of silica [26]. The sensor will be inadequate in the non-wetting condition, when the liquid is repelled from the cavity surfaces. Note that the fluid attributes which govern the evaporation dynamics do not relate directly to its refractive index or its absorption spectrum. Therefore, the measurement principle can add a new and complementary perspective to the fiber-optic sensing of liquids, beyond those of more traditional approaches. For example, the refractive indices of ethanol and acetone at 1,550 nm wavelength differ by less than 0.003. The differences between their evaporation dynamics, as seen in Figure 9, are large and easily detected.

Further research of the proposed sensors may follow several potential directions. First, the sensitivity limitations in the recognition of liquids and mixtures need to be quantified. To that end, advanced algorithms for the clustering of data can be employed [27]. Next, several MCs can be cascaded to provide a quasi-distributed monitoring of evaporation from a large number of points. Last, various coating and surface treatments, such as the deposition of functionalized self-assembled monolayers [28], could be used to modify the wetting behavior and droplet geometry of specific fluids of interest, and assist in their identification.

Potential specific applications of the proposed sensors could include the following: (a) simple alternative to direct observation of droplets in research laboratories; (b) remote monitoring of precipitation in climatology; (c) monitoring chemical reactions such as the production of bio-diesel by microwaves; (d) quality control of water, beverages and oils; (e) monitoring gasoline dilutions and quality, in harsh environments such as at the gas station or inside fuel tanks; (f) mobile point-of-care diagnostics, and other similar applications.

## Figures and Tables

**Table 1.** Geometric profiles of droplets used in simulations.

**Profile Type**	***X***(***z*, *t***)
Linear	$X_{1}=\left|O\left(t\right)-H\right|\left|1-\frac{2z}{L}\right|-O\left(t\right)$
Parabolic	$X_{2}={\left(\frac{2}{L}\right)}^{2}\;\left(H-O(t)\right)\left(z^{2}-Lz\right)+H$
